# Vitamin D status, dietary intake, and bone turnover in female Soldiers during military training: a longitudinal study

**DOI:** 10.1186/1550-2783-9-38

**Published:** 2012-08-06

**Authors:** Laura J Lutz, J Philip Karl, Jennifer C Rood, Sonya J Cable, Kelly W Williams, Andrew J Young, James P McClung

**Affiliations:** 1 Military Nutrition Division, United States Army Research Institute of Environmental Medicine, Natick, MA 01760, USA; 2 Pennington Biomedical Research Center, Louisiana State University System, Baton Rouge, LA 70808, USA; 3 Directorate of Basic Combat Training, Fort Jackson, SC 29207, USA

**Keywords:** Vitamin D, Calcium, Parathyroid hormone, Bone turnover, Soldiers

## Abstract

**Background:**

Vitamin D is an essential nutrient for maintaining bone health, to include protecting against stress fracture during periods of rapid bone turnover. The objective of this longitudinal, observational study was to assess vitamin D status, biomarkers of bone turnover, and vitamin D and calcium intake in female Soldiers (n = 91) during US Army basic combat training (BCT).

**Methods:**

Anthropometric, biological and dietary intake data were collected at wk 0, 3, 6, and 9 of the 10 wk BCT course. Mixed models repeated measures ANOVAs were used to assess main effects of time, race, and time-by-race interactions.

**Results:**

White volunteers experienced a decrease in serum 25(OH)D levels, whereas non-white volunteers experienced an increase during BCT. However, serum 25(OH)D levels were lower in non-whites than whites at all timepoints (P-interaction < 0.05). Group mean PTH levels increased (P < 0.05) during the first 3 wk of training, remained elevated for the duration of BCT, and were higher in non-whites compared to whites (P-race < 0.05). Biomarkers of both bone formation (bone alkaline phosphatase and procollagen I N-terminal peptide) and resorption (tartrate-resistant acid phosphatase and C-terminal telopeptide) increased (P < 0.05) during BCT, indicating increased bone turnover. Estimated daily intakes of vitamin D and calcium were below recommended levels (15 μg and 1000 mg/day, respectively), both before (group mean ± SEM; 3.9 μg/d ± 0.4 and 887 mg/d ± 67) and during BCT (4.1 μg/d ± 0.3 and 882 mg/d ± 51).

**Conclusions:**

These findings demonstrate that female Soldiers experience dynamic changes in vitamin D status coupled with increased bone turnover and potentially inadequate vitamin D and calcium intake during military training.

## Background

Vitamin D is an essential nutrient for the maintenance of human health and performance. Various biological roles have been described for vitamin D, including cardiac, immune, and musculoskeletal functions [1,2]. Perhaps the best described function of vitamin D is as an endocrine regulator of calcium homeostasis. The biologically active form of vitamin D, 1,25-dihydroxyvitamin D (1,25(OH)_2_D), affects intestinal calcium absorption by inducing the synthesis of the calcium transport protein calbindin [3]. Low 1,25(OH)_2_D levels diminish intestinal calcium absorption and induce parathyroid hormone (PTH) secretion. PTH stimulates resorption of calcium from bone in an effort to maintain serum calcium levels [4]. Diminished vitamin D status may degrade bone health, and has been associated with osteomalacia in adults [5], and low bone mineral content (BMC) and bone mineral density (BMD) in children and adults [6].

Poor vitamin D status may increase stress fracture risk [7,8]. Stress fractures are more prevalent in females than males. It has been estimated that up to 20% of female athletes and military personnel may experience a stress fracture during training [9]. Suboptimal vitamin D status (assessed using serum 25-hydroxyvitamin D (25(OH)D levels) may contribute, as military training may affect biomarkers of both bone formation and resorption [10], and declines in serum (25(OH)D) levels have been observed in female personnel undergoing military training [11]. Further, supplementation with 20 μg of vitamin D in conjunction with 2000 mg of calcium reportedly reduced stress fracture incidence in female Navy recruits [12].

Despite observations of diminished serum 25(OH)D levels during military training, and the elevated risk of stress fracture in female military personnel [13], no study has comprehensively assessed the effects of military training on serum 25(OH)D, PTH levels and biochemical indices of bone turnover in female Soldiers. Similarly, dietary intake of vitamin D and calcium have not been assessed during military training. As such, the objective of this longitudinal, observational study was to assess the effects of US Army basic combat training (BCT) on serum 25(OH)D, PTH levels, bone turnover, and vitamin D and calcium intake in female Soldiers. Effects of race on outcome measures were also assessed, as racial differences in serum 25(OH)D levels have been described previously by our group [11] and others [14,15]. We hypothesized that vitamin D status would improve in Soldiers training during the early spring months in the Southeastern US, as solar load increases in this location during the early spring, and that indicators of both bone formation and resorption would be increased in response to the physical activity experienced during military training.

## Methods

### Participants

This study was approved by the Human Use Review Committee at the United States (US) Army Research Institute of Environmental Medicine and was conducted at Fort Jackson, SC between the months of February and April. Human volunteers participated in this study after giving their free and informed consent. Investigators adhered to US Army Regulation 70–25 and US Army Medical Research and Material Command regulation 70–25 on the participation of volunteers in research.

The data provided in this report were collected as a part of a larger study assessing cardiometabolic risk in military recruits [16]. A total of 91 female Soldiers consented to participate in the present study. Body composition and demographic data were collected within one wk of the start (baseline) and completion (wk 9) of BCT. Hematological data were collected at four timepoints through BCT; at baseline and wk 3, 6, and 9. A total of 71 female Soldiers were included in the statistical analysis; volunteers were excluded from statistical analysis if they withdrew from the study, separated from the Army or their baseline or wk 9 data were missing. Demographic characteristics of the volunteers appear in Table 1.

**Table 1:** Female volunteer characteristics at baseline*

	**Group (n = 71)**	**White (n = 45)**	**Non-white (n = 26)**
Age, yr	23.1 ± 0.7	23.5 ± 1.0	22.4 ± 0.9
Height, cm	162.7 ± 0.7	163.1 ± 0.8	162.2 ± 1.3
Weight, kg	66.1 ± 1.0	64.9 ± 1.3	68.1 ± 1.4
BMI, kg/m^2^	24.9 ± 0.3	24.4 ± 0.4	25.9 ± 0.4†
Body Fat,%	26.6 ± 0.7	25.2 ± 0.8	28.9 ± 1.0
Race, n			
White or Caucasian	45		
Black or African American	18		
Asian	1		
Other	7		

* Mean ± SEM; † Different from white (P < 0.05).

### Basic combat training

The BCT course is the initial exposure to military training for individuals who enlist in the US Army. It is a 9–10 wk course that consists of both outdoor and indoor classroom training [17]. However, during most portions of the training, Soldiers wear combat uniforms which allow exposure of only the hands, neck, and face to the sun. Physical training is conducted outdoors and is comprised of aerobic (i.e., road marching, navigating obstacle courses, and running) and strength-training activities (i.e., calisthenics, push-ups, and sit-ups). The BCT course typically results in increased ambulatory activity; previously collected data regarding energy expenditure during BCT have been reported elsewhere [18].

The feeding environment at BCT consists of ad libitum cafeteria-style meals for breakfast, lunch, and dinner. Foods offered meet military dietary reference intakes (MDRIs) [19], which are similar to the DRIs for the American population, but adjusted for the specific needs of the military. Food offererings at military dining facilities aim to provide a well balanced diet and meet the Dietary Guidelines for Americans [19].

### Anthropometric measures

Weight was measured and recorded to the nearest 0.01 kg on a calibrated digital scale (A&A Scales, Prospect Park, NJ), and height was measured to the nearest 0.01 cm with a stadiometer (Creative Health Products, Plymouth, MI). Body fat percentages were estimated from skinfold thicknesses. Skinfold measurements were recorded using Lange calipers (Beta Technology, Santa Cruz, CA) at the triceps, suprailiac, and abodominal sites, and were rounded to the nearest 1.0 mm. Body density was calculated according to the 3-site skinfold equation for women [20], and body fat percentage was then determined using sex-, age-, and race-specific calculations [21].

### Biological samples

After an overnight fast, blood was collected from rested volunteers through antecubital venipuncture, processed on site, frozen, and shipped to the Pennington Biomedical Research Center (Baton Rouge, LA) for processing. Serum 25(OH)D levels (DiaSorin Inc., Stillwater, MN) were determined using a commercially available radioimmunoassay and PTH levels (Siemens 2000, Los Angeles, CA) were determined using a commercially available immunoassay. Serum bone alkaline phosphatase (BAP; Octeia, Fountain Hills, AZ), procollagen I N-terminal peptide (PINP; Orion Diagnostica, Espoo, Finland), tartrate-resistant acid phosphatase (TRAP; Immunodiagnostics Systems, Fountain Hills, AZ), and C-terminal telopeptide (CTx; Immunodiagnostics Systems, Fountain Hills, AZ) were determined using immunoassays. Serum IL-6 concentrations were determined using a multiplex assay with a lower detectible limit of 3.2 ng/L (Milliplex MAP; Millipore, Billerica, MA) and high-sensitivity C-reactive protein (hsCRP) concentrations were determined with an automated immunoassay instrument with a lower detectible limit of 0.2 mg/L (Siemens Medical Solutions USA, Inc.).

### Dietary intake

Self-reported dietary intakes of vitamin D and calcium before and during BCT were determined using a full-length, quantitative food frequency questionnaire (FFQ) (Block 2005 FFQ; NutritionQuest, Berkeley, CA). The FFQ was administered at baseline and wk 9 to estimate usual dietary intake from all food groups over the 3 mo prior to beginning training and during the 10-wk training course. Mean daily intakes of vitamin D and calcium were calculated from the USDA Food and Nutrient Database for Dietary Studies v. 1.0. Dietary supplements are not permitted during BCT.

### Statistical analysis

Statistical analyses were performed using the Statistical Package for the Social Sciences v. 18.0 (SPSS Inc., Chicago, IL). Descriptive statistics are presented as mean ± SEM. Normality was assessed using the Kolmogorov-Smirnov test. Race was treated as a dichotomus variable (white (n = 45) or non-white (n = 26)). Mixed models repeated measures ANOVA with race and time included as fixed variables, and participant treated as a random variable, was used to assess main effects of time and race as well as time-by-race interactions. Akaike’s information criteria were used to determine appropriate covariance structures. When a significant time-by-race interaction was observed, all possible t-tests with Bonferroni corrections were used to identify differences within and between groups. Log transformed variables were used in mixed models repeated measures ANOVA for variables that did not follow a normal distribution. Pearson’s or Spearman’s rank correlation were used as appropriate to test for associations between 25(OH)D levels and markers of inflammation (hsCRP and IL-6) and measures of body composition (body mass index (BMI) and body fat percentage). Mean daily intakes of vitamin D and calcium were compared to the US recommended dietary allowance (RDA) to compare experimental observations and population recommendations.

## Results

### Vitamin D status, PTH, and bone turnover

Serum 25(OH)D levels during BCT decreased 8% in whites but increased 21% in non-whites (*P*-interaction < 0.05, Table 2). At all time points, serum 25(OH)D levels were lower in non-whites than whites (*P*-interaction < 0.05). Group mean PTH increased within 3 weeks, and then remained elevated for the duration of BCT (*P*-effect < 0.05, Table 2). Mean PTH levels were greater in non-whites than whites (*P*-effect < 0.05).

**Table 2:** Longitudinal changes in serum 25(OH)D and PTH levels in female Soldiers during BCT*

	**Baseline**	**Wk 3**	**Wk 6**	**Wk 9**	**Effect**
25(OH)D, *nmol/L*					T x R
Group (n = 71)	64.1 ± 3.8	60.4 ± 2.9	60.7 ± 2.6	63.2 ± 2.6	
White (n = 45)	77.0 ± 3.5	70.6 ± 3.5†	68.6 ± 3.5†	70.5 ± 3.5	
Non-white (n = 26)	41.7 ± 4.6§	42.6 ± 4.6§	47.8 ± 4.6§	50.6 ± 4.6‡,§	
PTH, p*g/mL*					T, R
Group (n = 71)	32.7 ± 1.7	40.0 ± 1.7†	43.8 ± 1.8†	42.3 ± 2.2†	
White (n = 45)	31.9 ± 2.3	36.7 ± 2.3	39.7 ± 2.3	38.6 ± 2.3	
Non-white (n = 26)	34.0 ± 3.0	45.7 ± 3.1	50.7 ± 3.0	48.8 ± 3.0	

*Mean ± SEM; † Different from baseline (P < 0.05); ‡Different from week 3 (P < 0.05); §Different from white, (P < 0.05); T, main effect of time (P < 0.05); R, main effect of race (P < 0.05); T x R, time-by-race interaction (P < 0.05).

Markers of bone formation, BAP and PINP, and bone resorption, TRAP and CTx, increased (*P*-effect < 0.05, Table 3) during BCT. There was no differential effect of race on markers of either bone formation or resorption.

**Table 3:** Longitudinal changes in bone biomarkers in female Soldiers during BCT*

	**Baseline**	**Wk 3**	**Wk 6**	**Wk 9**	**Effect**
*Bone Absorption Biomarkers*					
BAP, *μg/L*					T
Group (n = 71)	27.6 ± 1.6	36.6 ± 1.9^†^	39.1 ± 1.9^†^	38.8 ± 2.0^†^	
White (n = 45)	26.2 ± 2.3	33.9 ± 2.4	37.1 ± 2.3	36.9 ± 2.3	
Non-white (n = 26)	29.9 ± 3.0	41.1 ± 3.1	42.9 ± 3.1	42.0 ± 3.0	
PINP, *μg/L*					T
Group (n = 71)	62.4 ± 3.7	75.1 ± 3.8†	78.7 ± 3.8†	78.7 ± 3.7†	
White (n = 45)	62.9 ± 4.5	72.5 ± 4.6	75.1 ± 4.5	77.7 ± 4.5	
Non-white (n = 26)	61.9 ± 5.9	77.7 ± 6.0	82.4 ± 6.0	79.8 ± 5.9	
*Bone Resorption Biomarkers*					
TRAP, *U/L*					T
Group (n = 71)	4.3 ± 0.2	4.6 ± 0.2	4.8 ± 0.2†	5.0 ± 0.2†, ‡	
White (n = 45)	4.2 ± 0.2	4.7 ± 0.2	4.8 ± 0.2	5.0 ± 0.2	
Non-white (n = 26)	4.5 ± 0.3	4.4 ± 0.3	4.8 ± 0.3	5.0 ± 0.3	
CTx, *μg/L*					T
Group (n = 71)	1.1 ± 0.1	1.0 ± 0.1	1.2 ± 0.1	1.2 ± 0.1‡	
White (n = 45)	1.2 ± 0.1	1.1 ± 0.1	1.1 ± 0.1	1.2 ± 0.1	
Non-white (n = 26)	1.0 ± 0.1	1.0 ± 0.1	1.2 ± 0.1	1.1 ± 0.1	

*Mean ± SEM; †Different from baseline (P < 0.05); ‡Different from week 3 (P < 0.05); T, main effect of time (P < 0.05).

### Anthropometrics and associations with vitamin D Status

No significant correlations were noted between 25(OH)D levels or biomarkers of inflammation at either baseline or wk 9 (data not shown). Similarly, no significant correlations between 25(OH)D levels and body fat percentage or BMI were documented at baseline in the total study population. In non-whites, however, there was a positive correlation between body fat percentage and 25(OH)D levels at baseline (0.46; *P* < 0.05).

### Vitamin D and calcium intake

**Figure 1:**
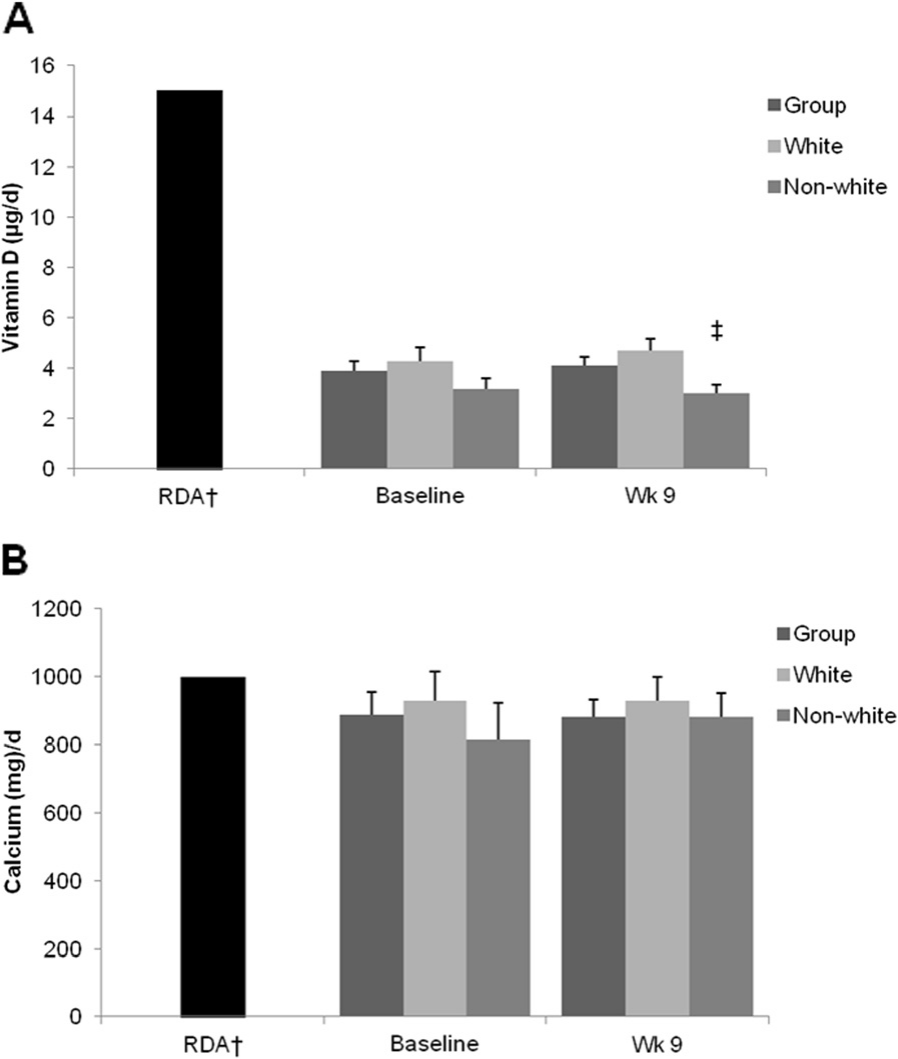
Reported vitamin D and calcium intake before and during BCT * *Mean ± SEM; n =71 (white = 45, non-white = 26); †RDA for women 19–30 years of age (Institute of Medicine, 2011); ‡Different from white, P <0.05.

In the total study population, reported mean daily intakes of vitamin D and calcium were below current RDA levels [22] both before and during BCT (Figure 1). Whites reported consuming more vitamin D during BCT when compared to non-whites (*P* < 0.05). Neither reported vitamin D nor calcium intake changed during the course of BCT, regardless of race.

## Discussion

The objective of this longitudinal, observational study was to assess the effects of military training on serum 25(OH)D, PTH levels, bone turnover, and vitamin D and calcium intake in female Soldiers during BCT. The major finding was a differential response of serum 25(OH)D during BCT: 25(OH)D levels declined in white volunteers, but increased in non-white volunteers. Serum 25(OH)D levels were greater in white volunteers than non-white volunteers throughout BCT. Additionally, military training resulted in significant increases in PTH and markers of both bone formation and resorption, regardless of race. Estimated dietary intakes of vitamin D and calcium did not meet current RDAs, either before or during BCT. These data confirm earlier findings demonstrating a decline in 25(OH)D levels in white female Soldiers during military training [11], and indicate that non-white Soldiers respond differently. Further, the data suggest that military training results in increased bone turnover and may elevate calcium demands in female Soldiers, as demonstrated by the increases in PTH observed in this study cohort.

Suboptimal vitamin D status, coupled with the unaccustomed physical activities associated with military training, may have profound effects on bone health. During bone remodeling, resorption and formation are coupled; however, once resorption occurs, bone deposition may require up to 90 days for completion [23], and may induce temporary weaknesses at remodeling sites. Evans et al. [10] noted increases in both markers of bone formation and resorption during military training, similar to the findings of the present study. Similarly, studies assessing the effects of resistance-type training have documented increases in markers of bone formation, and a reduction in markers of bone resorption [24]. The increase in markers of both bone resorption and formation observed in the present study may indicate a mechanism to repair microdamage caused by repeated stress. If stress continues to affect bone, microdamage may further develop into stress fractures.

Stress fracture is of particular concern in military personnel, as up to 60% of female Soldiers that experience fracture may attrite from military training [12,25,26]. Studies reviewing stress fracture risk in military personnel indicate that a number of factors not affected by diet, such as female sex, menstrual status, contraceptive use, or polymorphisms in the vitamin D receptor, may be strong predictors of fracture risk [8,12,25]. Other factors, such as optimizing vitamin D status, may provide the opportunity to limit fracture risk through intervention. For example, Ruohola et al. [7] found that serum levels of 25(OH)D below the study population median (76 nmol/L) at the onset of military training was a significant risk factor for stress fracture in Finnish male military personnel. Burgi et al. [14] confirmed the relationship between 25(OH)D levels and stress fracture risk; in a case–control study with female Navy recruits it was determined that stress fracture risk was approximately double in volunteers who began training in the lowest quintile of 25(OH)D levels (35 nmol/L) as compared to those in the top quintile (124 nmol/L).

In a recent randomized, placebo-controlled intervention trial, Lappe et al. [12] found that daily provision of supplements containing 20 μg of vitamin D and 2000 mg of calcium reduced stress fracture incidence by up to 20% in female Navy recruits during training. Although this nutritional intervention appears beneficial for the prevention of stress fracture, the study did not include biochemical or functional assessments of serum 25(OH)D levels, PTH or bone health. As such, it is difficult to draw definitive conclusions regarding the mechanism by which supplementation with vitamin D and calcium may have conferred protection. Comprehensive intervention trials including the collection of observational data regarding stress fracture occurrence coupled with biochemical and functional indicators of nutritional status and bone health will be required to determine the true efficacy of vitamin D and calcium interventions for the prevention of stress fracture in military personnel.

Our data indicate significant racial differences in serum 25(OH)D levels. For example, mean levels of serum 25(OH)D were greater in white volunteers as compared to non-white volunteers at the start of training. Further, serum 25(OH)D levels increased in non-whites, but declined in white volunteers over the course of the training period. Racial differences in serum 25(OH)D levels have been described previously by our group [11] and others [15,27]. Paradoxically, although non-white populations tend to have lower mean serum 25(OH)D levels than white populations, non-white populations are at reduced risk for both osteoporotic [28,29] and stress fractures [25]. Racial differences in the relationship between vitamin D status and bone health may be due to a number of factors, including differences in BMD [30,31] and bone geometry [30-32]. Other factors may include sensitivity to PTH. Skeletal resistance to PTH-stimulated bone resorption has been described in non-white populations [33], and may provide a mechanism by which non-white populations with suboptimal serum 25(OH)D levels retain BMD. In the present study, both serum 25(OH)D and PTH levels increased in non-white volunteers during training. In contrast, serum 25(OH)D levels declined in white volunteers during BCT as levels of PTH increased. This finding indicates racial differences in the relationship between serum 25(OH)D and PTH levels during military training, and warrants further scientific exploration, to include factors not assessed in the present study, such as the influence of physical activity and sunlight exposure.

Recent studies have used serum 25(OH)D cutoff values as indicators of suboptimal vitamin D status in populations. Some have recommended cutoff values of ≤75 nmol/L [34,35]. Using this cutoff value to define inadequacy, 64% and 92% of white and non-white volunteers in this study completed BCT with suboptimal vitamin D levels, respectively. The most recent Institute of Medicine report on DRIs for calcium and vitamin D [22] is less conservative, suggesting that individuals may be at risk of vitamin D deficiency relative to bone health at serum 25(OH)D values ≤30 nmol/L. Applying this cutoff value, no white volunteers and 8% of non-white volunteers completed BCT with suboptimal 25(OH)D levels. However, it is possible that the increased bone turnover experienced during BCT may affect the vitamin D requirement for this subpopulation. Data gleaned from this study and others [10] indicate increases in markers of both bone absorption and resorption during military training indicative of increased bone turnover. Increasing levels of PTH may suggest elevated calcium demand during training and may affect the vitamin D requirement in populations experiencing periods of rapid bone turnover.

Our data indicate that dietary consumption of vitamin D and calcium did not meet current recommendations [22] both before and during BCT. In fact, volunteers consumed less than one third of the current RDA for vitamin D both before and during training. Although sunlight exposure was not quantified during BCT, declines in serum 25(OH)D levels observed in white volunteers coupled with suboptimal serum 25(OH)D levels in non-white volunteers throughout the study indicate that strategies to improve dietary intake of vitamin D and calcium during military training may be needed to improve vitamin D status. Further, sweat mineral losses were not quantified in the present study. Estimates of mineral losses through sweat vary depending upon collection and assay techniques [36-38]. If significant calcium losses were to occur through sweating during military training, this could affect nutritional requirements and could affect bone health by stimulating PTH [39].

## Conclusion

In summary, this longitudinal study determined vitamin D status during military training in females, to include interactions between vitamin D status and race. Serum 25(OH)D levels declined in white volunteers, and were lower in non-white volunteers as compared to white volunteers at all timepoints. Increases in PTH and indicators of bone turnover were observed during military training. Our findings indicate that efforts to improve the dining environment during military training should emphasize the consumption of foods containing vitamin D and calcium, as the cohort of Soldiers participating in the present study did not meet current recommended intakes for either nutrient. Strengths of the study included the longitudinal design in an environment free of dietary supplements and other factors that may have affected the carefully controlled collection of dietary status and intake data. Weaknesses include the lack of functional data regarding bone health and injury outcomes and a lack of data quantifying sun exposure. Future studies should determine whether the increased PTH and bone turnover observed during military training affect the vitamin D requirement, and whether vitamin D and calcium supplementation may be prudent for the prevention of injury, to include stress fracture.

## Competing interest

LJL, JPK, JCR, SJC, KWW, AJY, and JPM, no conflicts of interest.

## Authors’ contributions

JPM and JPK designed research; JPK, SJC, KWW, and JPM conducted research; JCR processed biological samples; LJL and JPK conducted statistical analysis; LJL, AJY and JPM wrote the paper; JPM had primary responsibility for final content. All authors read and approved the final manuscript.
